# Absorbable and nonabsorbable packing after functional endoscopic sinus surgery: systematic review and meta-analysis of outcomes

**DOI:** 10.1007/s00405-014-3107-2

**Published:** 2014-06-14

**Authors:** Tang-Chuan Wang, Chih-Jaan Tai, Yung-An Tsou, Li-Tai Tsai, Yu-Fen Li, Ming-Hsui Tsai

**Affiliations:** 1 Department of Otolaryngology, China Medical University Hospital, Taichung, Taiwan; 2 Department of Otolaryngology-Head and Neck Surgery, University of Iowa, Iowa City, IA USA; 3 Department of Medicine, China Medical University, Taichung, Taiwan; 4 Graduate Institute of Biostatistics, China Medical University, Taichung, Taiwan

**Keywords:** Absorbable, Efficacy, Epistaxis, FESS, Functional endoscopic sinus surgery, Meta-analysis, Nasal, Nonabsorbable, Packing, Synechia

## Abstract

The purpose of the study was to perform a systematic review and meta-analysis of the literature to compare the efficacy (and other postoperative outcomes) of nonabsorbable versus absorbable nasal packing after functional endoscopic sinus surgery (FESS) for the treatment of chronic rhinosinusitis. Studies were considered for inclusion if they were published in English language, were randomized clinical trials, and reported on outcomes following postoperative synechia. The primary outcome for meta-analysis was the incidence of postoperative synechia; pooled odds ratios (ORs) and 95 % confidence intervals (CIs) were calculated using fixed-effects models. Five studies, involving 241 nasal cavities in each treatment group, were included in the systematic review. The prevalence of synechia ranged from 4.6 to 8.0 % in the absorbable groups and from 8.0 to 35.7 % in the nonabsorbable groups. Postoperative bleeding was lower in the absorbable groups, whereas there was no clear finding regarding postoperative pain. Postoperative edema was generally similar between groups. There were no consistent findings regarding bleeding and pain on packing removal. Two studies using the same type of packing material were included in the meta-analysis. The combined OR (0.33, 95 % CI 0.04–2.78) for postoperative synechia did not significantly favor (*P* = 0.308) absorbable packing over nonabsorbable packing. Although there is some evidence in the available literature that absorbable nasal packing may provide superior outcomes to nonabsorbable packing after FESS, the lack of homogeneity between studies makes definitive conclusions impossible. Further randomized clinical trials are needed to compare the efficacy of different types of absorbable nasal packing for preventing synechia after FESS.

## Introduction

Chronic rhinosinusitis is an extremely common condition, affecting millions of individuals the world over. Indeed, reports suggest that up to approximately 16 % of the adult population in the USA suffer from this condition [1, 2]. As chronic rhinosinusitis can have a significant negative impact on quality of life [3], treatment is typically required. In most cases, chronic rhinosinusitis can be managed through pharmacologic means; however, some individuals do not respond to such intervention and require surgery [2].

Functional endoscopic sinus surgery (FESS) is perhaps the most commonly used surgical approach for managing chronic rhinosinusitis [4, 5] and aims to improve/restore drainage and airflow throughout affected sinuses [2]. Although FESS is effective in more than 90 % of patients [6] and significantly improves quality of life [7], postoperative complications, in particular bleeding and adhesions (synechia), are not uncommon [8]. As a consequence, the nasal cavity is often packed after FESS with material designed to stem any ongoing bleeding, reduce clot formation, ameliorate the risk of synechia, and promote healing [8, 9]. Traditionally, nonabsorbable nasal packing has been applied after FESS [7]; however, such packing, and subsequent removal, is not well tolerated by patients [10]. More recently, absorbable nasal packing has been introduced and appears to be well tolerated by patients [11, 12].

Although a number of studies have compared the efficacy of nonabsorbable and absorbable nasal packing after FESS [8–14], there is conflicting evidence between studies as to whether one method is superior to the other or whether the methods have comparable efficacy. Therefore, we performed a systematic review and meta-analysis of the available literature in an effort to gain a better understanding of the efficacy and other outcomes concerning nonabsorbable versus absorbable nasal packing after FESS for the treatment of chronic rhinosinusitis. We included randomized trials only and examined synechia in our meta-analysis as a key indicator of nasal packing efficacy.

## Materials and methods

### Search strategy

MEDLINE, Current Contents, and the Cochrane databases were searched on January 31, 2013, using combinations of the following search terms: FESS, rhinosinusitis, bleeding, gelatin, hyaluronic acid, carboxymethylated cellulose (CMC), and packing.

### Selection of studies

Studies were considered for inclusion in the systematic review and meta-analysis if they were available in English, were randomized clinical trials, and reported on postoperative pain, edema, synechia/adhesion, and/or bleeding/hemostasis as study outcomes. Studies that did not meet these criteria were excluded.

### Data extraction

Data were extracted by two independent reviewers who consulted with a third reviewer, as necessary, to resolve any disagreements. For each eligible study, the following information and data were extracted: authors, year of publication, number of nasal cavities packed per treatment group, age of participants, sex distribution of participants, the type of nasal packing used, postoperative treatment, the time to removal of packing, the incidence of postoperative synechia, the incidence of postoperative bleeding, postoperative pain, postoperative edema, and bleeding and pain on removal of packing.

The primary outcome for meta-analysis was the incidence of postoperative synechia for absorbable versus nonabsorbable nasal packing.

### Data analysis

Odds ratios (ORs) with 95 % confidence intervals (CIs) were calculated for binary outcomes and comparisons made for absorbable versus nonabsorbable nasal packing. A *χ* ^2^-based test of homogeneity was carried out, and the inconsistency index (*I* ^2^) statistic was determined. If *I* ^2^ was >50 % or >75 %, the studies were considered to be heterogeneous or highly heterogeneous, respectively. If *I* ^2^ was <25 %, the studies were considered to be homogeneous. If the *I* ^2^ statistic (>50 %) indicated heterogeneity existed between studies, a random-effects model was calculated. Otherwise, a fixed-effects model was calculated. Pooled summary statistics for ORs of the individual studies are reported. A *P* value <0.05 was taken to indicate statistical significance. All analyses were performed using Comprehensive Meta-Analysis statistical software, version 2.0 (Biostat, Englewood, NJ).

## Results

### Literature search

**Fig. 1:**
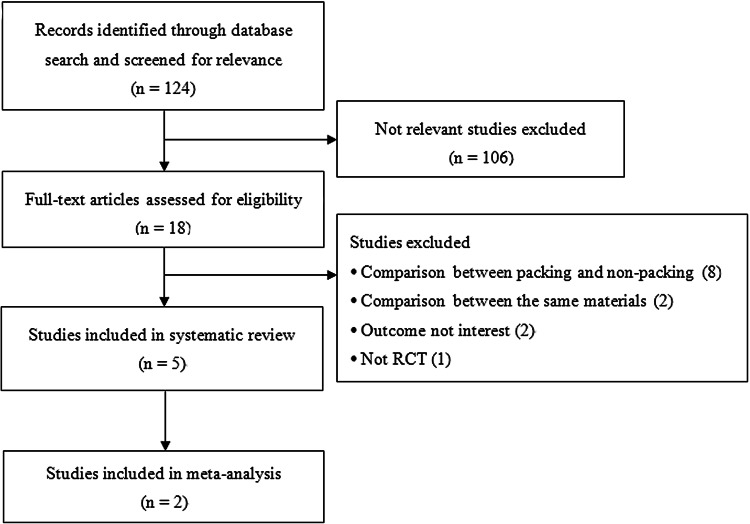
Flow diagram of study selection

A total of 124 records were retrieved in the database search (Fig. 1). Of these, 106 were excluded after title/abstract review, 13 were excluded after full-text review, and five were included in the systematic review (two of these studies were also included in the meta-analysis of postoperative synechia).

### Study characteristics

The characteristics of the studies [8, 11–13, 15] included in the systematic review are summarized in Table 1. The number of nasal cavities treated in each study ranged from 30 to 100 with a total of 241 nasal cavities treated in each group for all studies combined. The age of study participants was reported in four of the five studies [8, 11, 13, 15] and was generally similar among these studies, ranging from 35.7 to 43.2 years among three studies [8, 13, 15] and 54.0 years in one study [11]. The sex distribution of participants was also reported in the same four studies [8, 11, 13, 15], with the proportion of males ranging from 54 to 67 %. Regarding absorbable nasal packing materials, MeroGel^®^ was used in two studies [8, 12], while Cutanplast [15], CMC foam [13], and NasoPore [11] were used in one study each. Regarding nonabsorbable nasal packing material, Merocel was used in three studies [8, 11, 15] while polyvinyl alcohol sponges [12] and routine nasal packing (cotton gauze placed in a latex glove finger) [13] were used in one study each. Four of the five studies [8, 12, 13, 15] reported on postoperative treatments, all of which involved administration of various antibiotics. Three studies [8, 11, 13] reported on the time to packing removal, which ranged from 1 to 7 days.

**Table 1:** Characteristics of studies included in the systematic review

References	Nasal cavities packed, abs versus nonabs	Age (years)	Sex (male %)	Absorbable packing	Nonabsorbable packing	Postoperative treatment	Time to packing removal
Cho et al. [15]	100 versus 100	35.7	64	Cutanplast	Merocel	Second-generation cephalosporin or clarithromycin, analgesics as needed, prednisone	NA
Miller et al. [8]	37 versus 37	39.1	54	MeroGel^®^	Merocel	Cefuroxime, saline nasal spray and nasal irrigation	Postoperative day 5–7
Berlucchi et al. [12]	44 versus 44	NA	NA	MeroGel^®^	PVA sponge	Amoxicillin + clavulanic acid, nonaspirin analgesics as needed, saline nasal spray	NA
Szczygielski et al. [13]	30 versus 30	43.2	62	CMC foam	Routine packing^a^	Cefazolin sodium, decongestants	Postoperative day 1
Shoman et al. [11]	30 versus 30	54	67	NasoPore	Merocel	NA	Postoperative day 7

*Abs* absorbable nasal packing material, *CMC* carboxymethylated cellulose, *NA* data not available, *Nonabs* nonabsorbable nasal packing material, *PVA* polyvinyl alcohol

^a^Cotton gauze placed in a latex glove finger

### Study outcomes

The prevalence of synechia was reported in three studies [8, 12, 13] and ranged from 4.6 to 8.0 % in the absorbable packing groups and from 8.0 to 35.7 % in the nonabsorbable packing groups. The duration of follow-up for monitoring of postoperative synechia was 8 weeks in two studies [8, 13] and 12 weeks in one study [12]. Postoperative bleeding data were reported in two studies [11, 13], both of which found decreased bleeding in the absorbable group compared with the nonabsorbable group. Likewise, postoperative pain data were reported in the same two studies, one of which found that pain was considerable less in the absorbable group [13], whereas the other found that pain was less in the nonabsorbable group [11]. Three studies reported results on postoperative edema [8, 11, 12]. Two of these studies [8, 11] found no clear between-group differences in edema, whereas the other [12] found that edema was less pronounced in the absorbable group compared with the nonabsorbable group. Two studies [11, 15] each reported on bleeding and pain on packing removal. One study [15] found that pain and bleeding were both markedly reduced in the absorbable group compared with the nonabsorbable group, whereas the other study [11] found that pain and bleeding were similar between groups. The timing of the aforementioned assessments varied between studies (see Table 2).

**Table 2:** Summary of outcomes for studies included in the systematic review

References	Abs versus nonabs					
	Synechia	Postoperative bleeding	Postoperative pain	Postoperative edema	Bleeding on packing removal	Pain on packing removal
Cho et al. [15]	NA	NA	NA	NA	59 % versus 91 %	1.01 ± 0.16 versus 2.37 ± 0.19^a^
Miller et al. [8]	8.0 versus 8.0 % (8 weeks)	NA	NA	0.70 ± 0.45 versus 0.71 ± 0.45 (8 weeks)^f^	NA	NA
Berlucchi et al. [12]	4.6 versus 29.7 % (12 weeks)	NA	NA	43.2 versus 58.4 %	NA	NA
Szczygielski et al. [13]	6.7 versus 35.7 % (8 weeks)	13.3 % versus 6.7 %	5.5 (3–9) versus 0.962 (0–4) (24 h)^b^	NA	NA	NA
Shoman et al. [11]	NA	3.67 ± 2.45 versus 3.44 ± 2.01 (1st week)^d^	3.33 ± 2.50 versus 3.70 ± 2.98 (1st week)^d^	2.78 ± 2.52 versus 2.78 ± 2.36 (1st week)^d^	0.90 ± 0.55 versus 0.83 ± 0.53^e^	4.03 ± 2.80 versus 3.97 ± 2.72^d^

*Abs* absorbable nasal packing material, *Nonabs* nonabsorbable nasal packing material, *NA* data not available, *VAS* visual analog scale

^a^Five-point scale ranging from 0 to 4, where 0 = no pain and 4 = worst pain imaginable; ^b ^ VAS score ranging from 0 to 10, where 0 = no pain and 10 = worst pain imaginable; ^c ^pain greater than 4 on VAS; ^d^ subjective score ranging from 0 to 10, where 0 = no bleeding/pain/edema and 10 = maximal bleeding/pain/edema; ^e^ objective grade ranging from 0 to 3, where 0 = no bleeding and 3 = severe bleeding requiring repacking; ^f^ blinded edema score ranging from 0 to 3, where 0 = no visible mucosal edema and 3 = frank polyposis

### Quality assessment

The quality of the studies included in the systematic review was assessed as highlighted in Table 3. Comprehensive information was not available for all studies [8, 13], and outcome assessor, care provider, and/or patient blinding did not occur in several studies [8, 11, 12]. Otherwise, the studies generally had characteristics consistent with being high-quality trials. Of note, aside from not including an intention-to-treat analysis, the study reported by Cho et al. [15] met all of the quality criteria.

**Table 3:** Quality assessment of studies included in the systematic review

References	Method of randomization used	Groups similar at baseline regarding the most important prognostic indicators	Eligibility criteria specified	Outcome assessor blinded	Care provider blinded	Patient blinded	Point estimates and measures of variability presented for the primary outcome measures	Analysis included an intention-to-treat analysis
Cho et al. [15]	Y	Y	Y	Y	Y	Y	Y	N
Miller et al. [8]	Y	NA	Y	Y	N	N	Y	Y
Berlucchi et al. [12]	Y	Y	Y	Y	N	N	Y	Y
Szczygielski et al. [13]	Y	NA	Y	NA	NA	NA	Y	Y
Shoman et al. [11]	Y	Y	Y	Y	N	Y	Y	Y

*N* no, *NA* information not available or not applicable, *Y* yes

### Meta-analysis of postoperative synechia

**Fig. 2:**
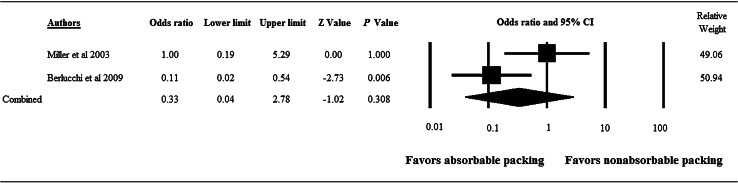
Forest plot showing OR for postoperative synechia after functional endoscopic sinus surgery with absorbable versus nonabsorbable nasal packing for the treatment of chronic rhinosinusitis. Data are presented as OR with 95 % CI. Heterogeneity test results: *Q* = 3.492, *df* = 1, *P* = 0.062, *I* ^2^ = 71.37 %

Note: meta-analysis of the other postoperative outcomes was not possible due to significance between study heterogeneity.

Two studies [8, 12] were included in the meta-analysis of synechia, the results of which are summarized in Fig. 2. There was significant heterogeneity between the two studies for this outcome (*Q* = 3.492, *I* ^2^ = 71.37 %, *P* = 0.062); therefore, a random-effects model of analysis was used. The combined OR for postoperative synechia did not significantly favor absorbable nasal packing over nonabsorbable nasal packing or vice versa (*P* = 0.308).

## Discussion

To our knowledge, this is the first systematic review/meta-analysis to compare outcomes (including efficacy as indicated by postoperative synechia) of absorbable versus nonabsorbable nasal packing after FESS for the treatment of chronic rhinosinusitis. A total of five randomized clinical trials, involving 241 nasal cavities in each treatment arm, met the criteria for inclusion in the systematic review. There was considerable variability in characteristics between studies, in particular, regarding the type of nasal packing material used. Postoperative bleeding was less with absorbable packing, whereas there were no between-group differences or consistent findings with regard to postoperative pain and edema, and pain and bleeding on packing removal. Of note, our meta-analysis, which included the findings from two studies, revealed that the incidence of postoperative synechia was not significantly reduced by absorbable compared with nonabsorbable nasal packing.

As already noted, our meta-analysis of results from randomized clinical trials revealed that absorbable nasal packing was not associated with a significantly lower risk of synechia after FESS for chronic rhinosinusitis compared with nonabsorbable nasal packing. Of the studies included in the systematic review part of the study, Szczygielski et al. [13] also reported on rates of synechia within 8 weeks of surgery and found a markedly lower rate among patients who received absorbable packing. Likewise, in a study not eligible for inclusion in our systematic review/meta-analysis, Hu et al. [16] found that there was a reduced rate of postoperative synechia among patients who received absorbable nasal packing (Meropack) compared with those who received no packing. In contrast, in a prospective, nonrandomized study, Baumann et al. [9] found little difference in the rate of postoperative synechia between patients who received absorbable (FloSeal) and nonabsorbable (Merocel) nasal packing. Several other studies have also failed to demonstrate any benefit of packing with CMC compared with no packing or nonabsorbable packing for reducing postoperative synechia [17, 18]. The disparate findings between studies clearly reflect the lack of homogeneity, most notably in the type of absorbable packing material used. Unfortunately, this lack of homogeneity restricted our ability to make any definitive conclusions. The variability in synechia outcomes between studies does, however, suggest that different types of absorbable packing materials are not created equal when it comes to reducing postoperative synechia. Clearly, further randomized trials are needed to directly compare the efficacy of different absorbable packing materials for reducing synechia after FESS for the treatment of chronic rhinosinusitis.

Only two studies included in our systematic review provided data on postoperative bleeding; however, both of these studies found decreased bleeding with absorbable packing. The findings from several previous studies also suggest that packing with absorbable material (Meropack, Gelfoam) reduces postoperative bleeding compared with no packing [16, 19]. Further, Jameson et al. [20] have also reported decreased postoperative bleeding after FESS in nasal passages packed with absorbable (FloSeal) compared with nonabsorbable packing. In contrast, several other studies have found no difference in postoperative bleeding with absorbable (NasoPore, CMC) versus nonabsorbable or no nasal packing [11, 21]. As with postoperative synechia, the lack of homogeneity between studies may explain the disparate findings. Additional randomized trials are needed to further investigate the efficacy of absorbable versus nonabsorbable nasal packing for preventing bleeding after FESS for the treatment of chronic rhinosinusitis.

In addition to postoperative synechia and bleeding, we also examined other outcomes after FESS, including postoperative edema and pain, and bleeding and pain on removal of packing. Unsurprisingly, there was again a lack of consistency in these results between studies, although it should be noted that the study reported by Cho et al. [15], which had the most number of patients and was the highest quality randomized controlled trial included (according to our assessment), did reveal markedly less bleeding and pain on removal of absorbable compared with nonabsorbable nasal packing.

Our study has a number of limitations that must be acknowledged. Firstly, both the type of packing material used and the duration of follow-up were different between several studies. This markedly restricted our ability to perform meta-analyses of results. Secondly, our analyses did not take into account other important factors that may have biased the study findings (and indeed our meta-analysis), including indicators of packing efficacy, such as postoperative infection and edema granulation, associated pathologies, such as nasal polyps, aspirin sensitivity, perioperative treatment, postoperative debridement, smoking history. Thirdly, we chose not to assess patient satisfaction as an outcome measure. Clearly, this is a very important consideration when evaluating the effectiveness of any treatment; however, we feel it is more important to conclusively determine which means of nasal packing is most clinically effective before considering patient satisfaction. We do note, however, that the results from a previous randomized controlled trial (not eligible for inclusion in our systematic review/meta-analysis) suggest that the majority of patients prefer absorbable nasal packing material (specifically MeroGel) over nonabsorbable material [10]. Fourthly, our meta-analysis only included a relatively small number of studies, thus limiting the power of analysis. Finally, we were not able to perform any analyses regarding the different types of FESS due to the lack of data/sufficiently detailed methodological descriptions.

The results of our systematic review and meta-analysis do not allow us to make any definitive conclusions regarding outcomes (including efficacy as indicated by the incidence of synechia) for the comparison of absorbable versus nonabsorbable nasal packing material after FESS. Clearly, there is some evidence to support the notion that absorbable packing may be superior to nonabsorbable packing; however, the distinct lack of homogeneity between studies reported in the current literature (particularly in the type of absorbable nasal packing material used) is a major limiting factor moving forward. Lack of homogeneity aside, our systematic review also highlights the fact that there is a scarcity of data available from high-quality randomized trials on the efficacy of absorbable versus nonabsorbable packing after FESS. Additional randomized controlled trials are needed, not only to provide more definitive information on the absorbable versus nonabsorbable packing debate, but also to compare the efficacy of different types of absorbable packing materials. We hope this report will help spur such trials.
